# A System of Anatomy for the Use of Students of Medicine

**Published:** 1839-07

**Authors:** 


ART. VIII.—*A System of Anatomy for the Use of Students of Medicine.* By CASPAR WESTON, M.D. *With Notes and Additions,* by W. E. HORNER, M.D. *Seventh Edition, entirely remodelled and illustrated by numerous Engravings,* by J. PANCOAST, M.D. &c. Two Vols.—*Philadelphia,* 1839. 8vo, pp. 491, 560.

THE numerous editions through which this work has run since its first publication in 1814 sufficiently attest the high value placed upon it in America. Although decidedly a work of merit, and well suited to elementary instruction, it is not calculated to supersede the many valuable manuals already in the hands of our students. The woodcuts are very indifferent, and the original plates commonplace and rather old-fashioned: the best illustrations are those copied from the elegant drawings of Sir Charles Bell.

